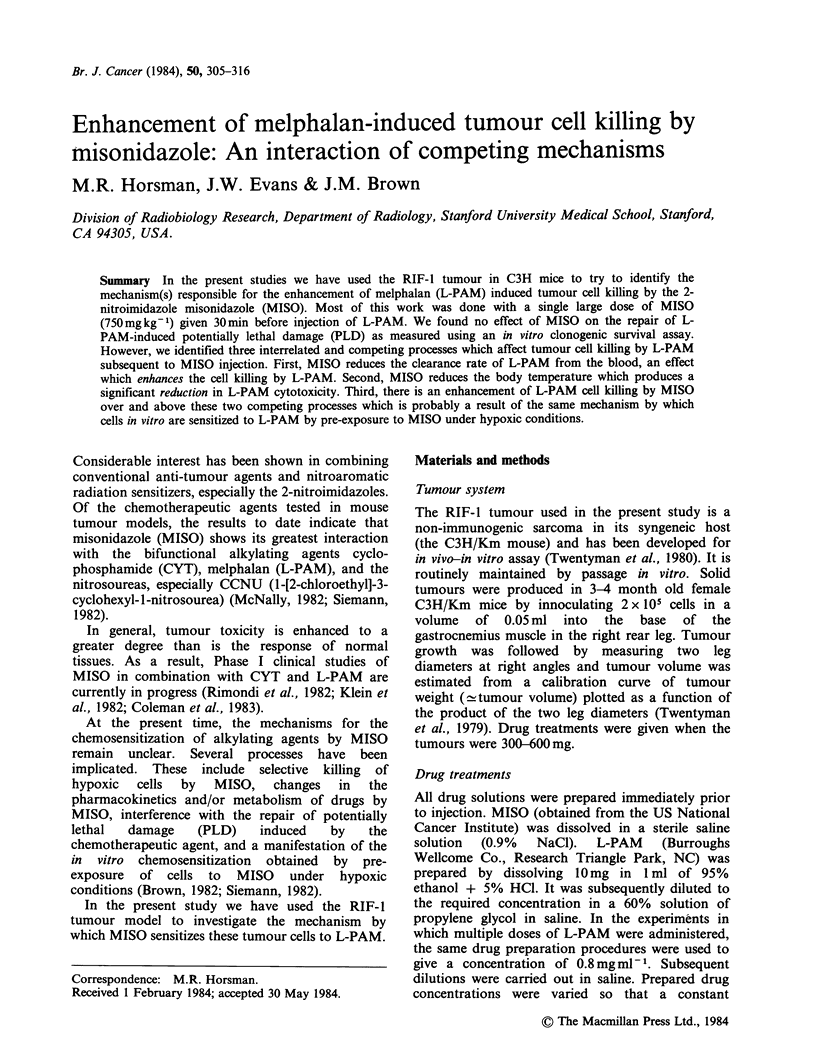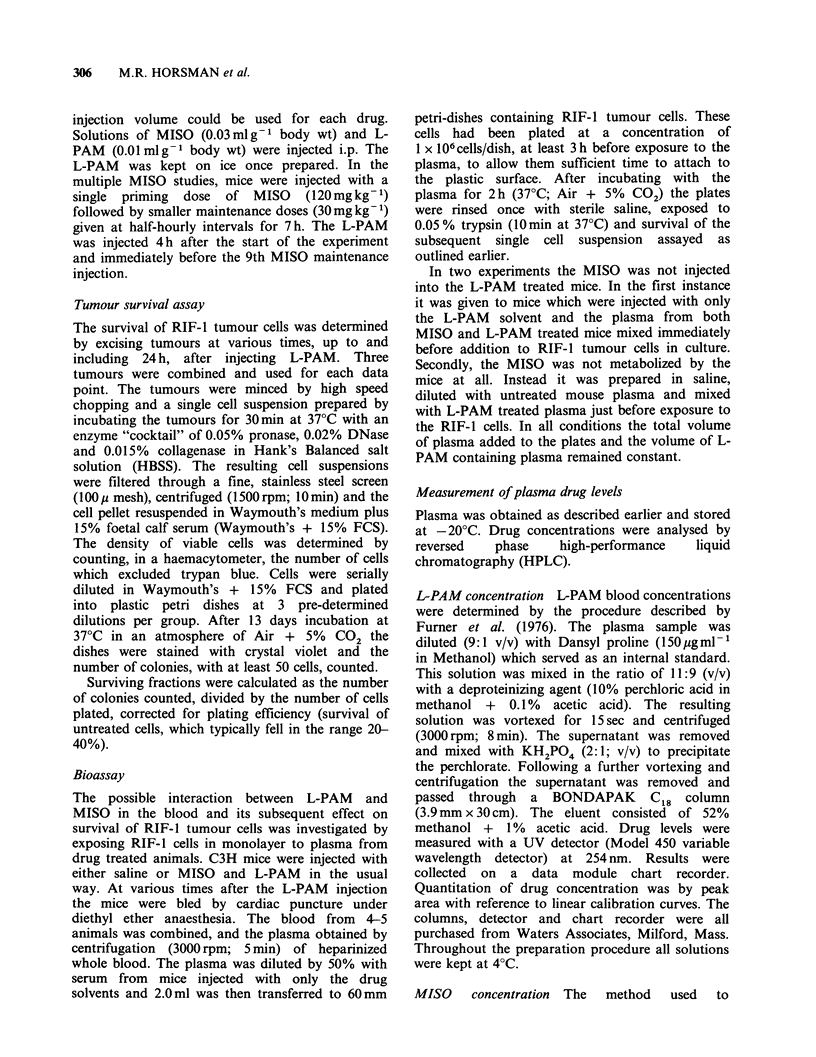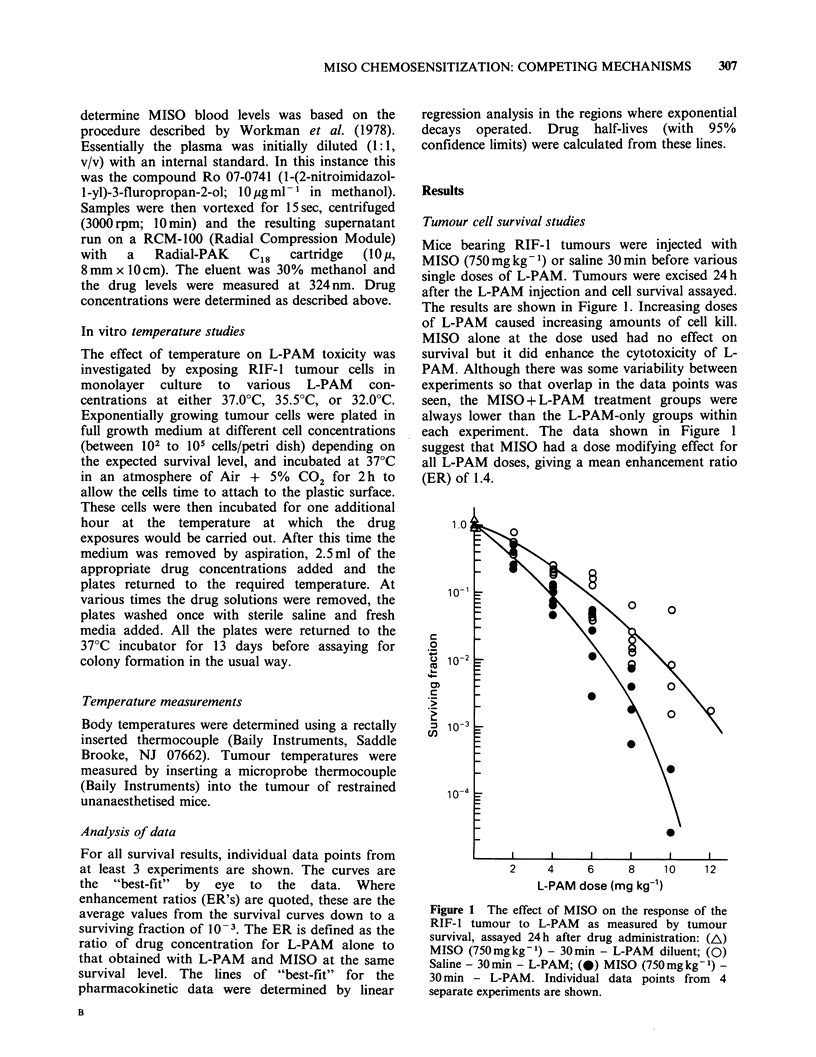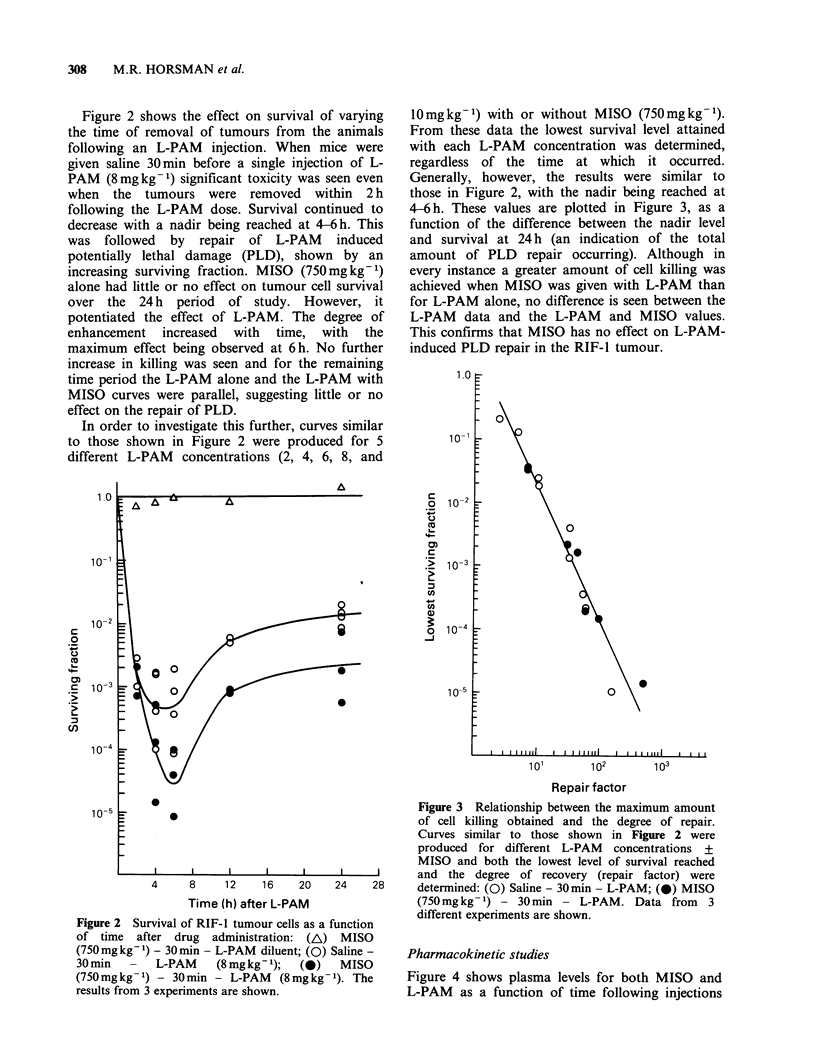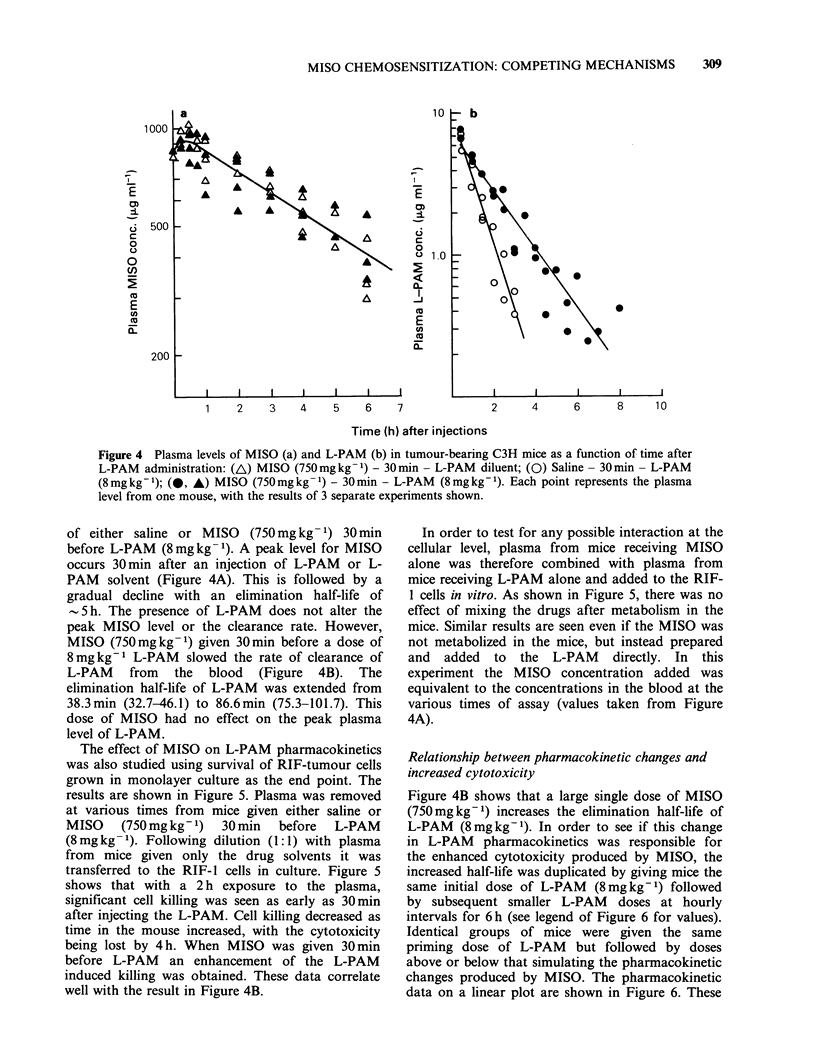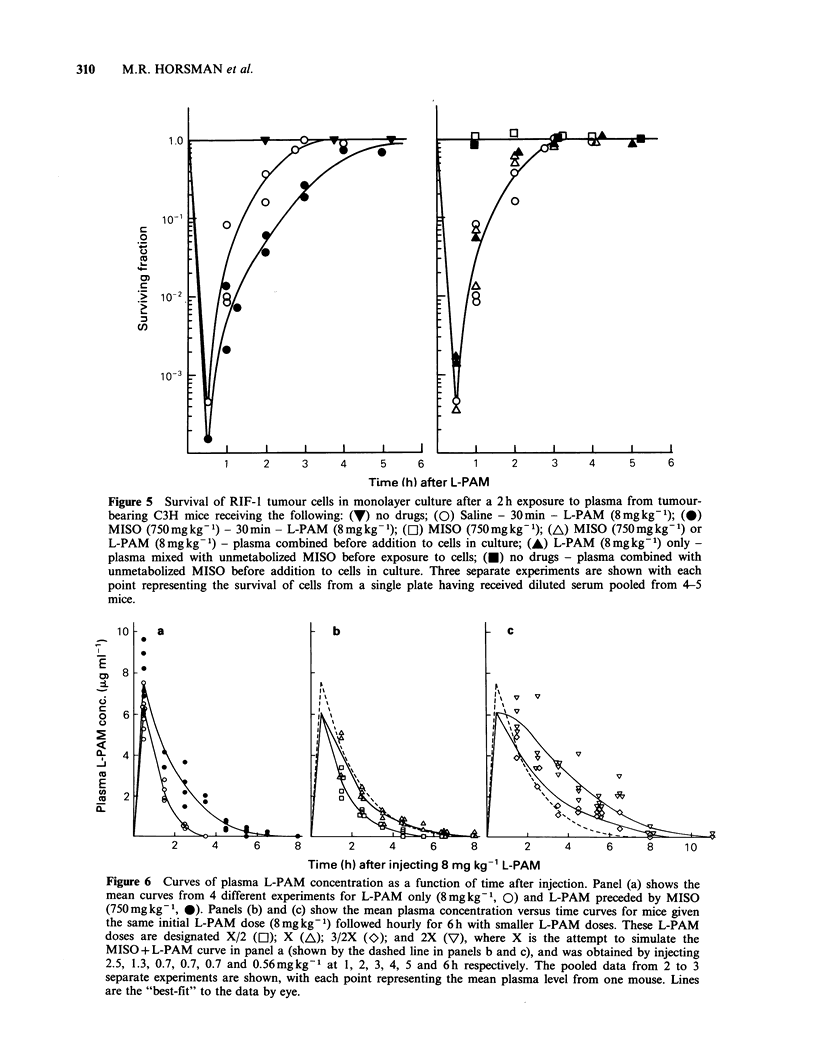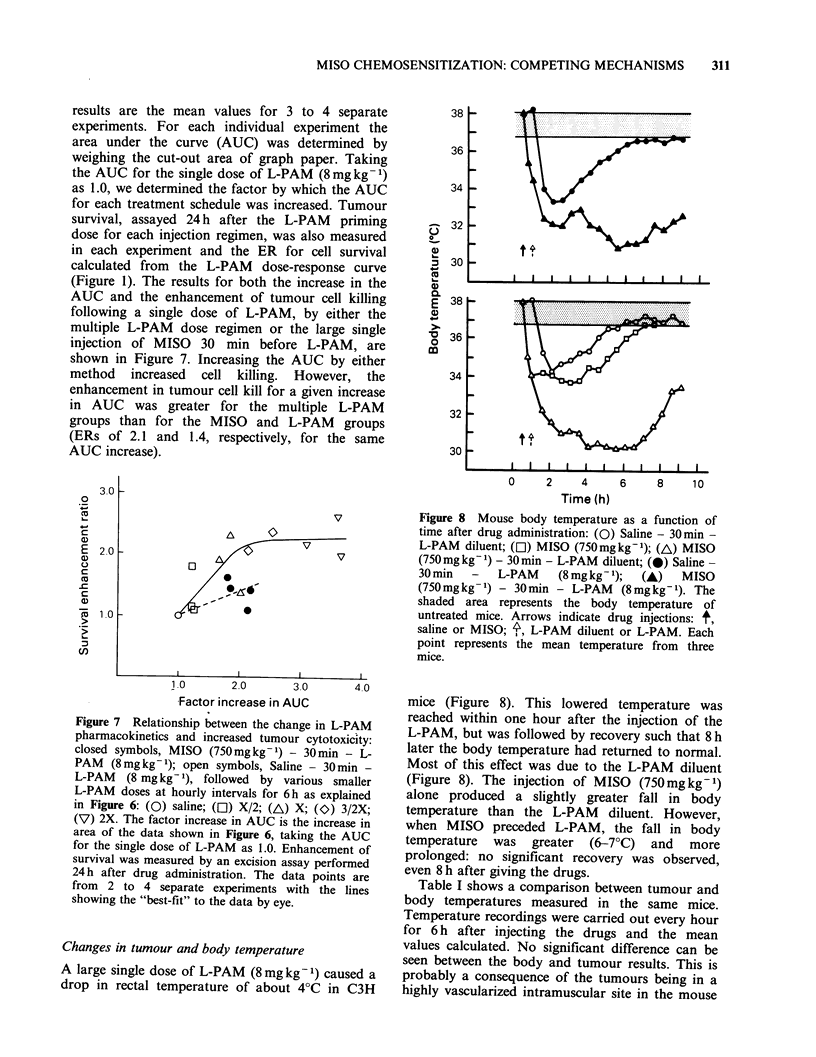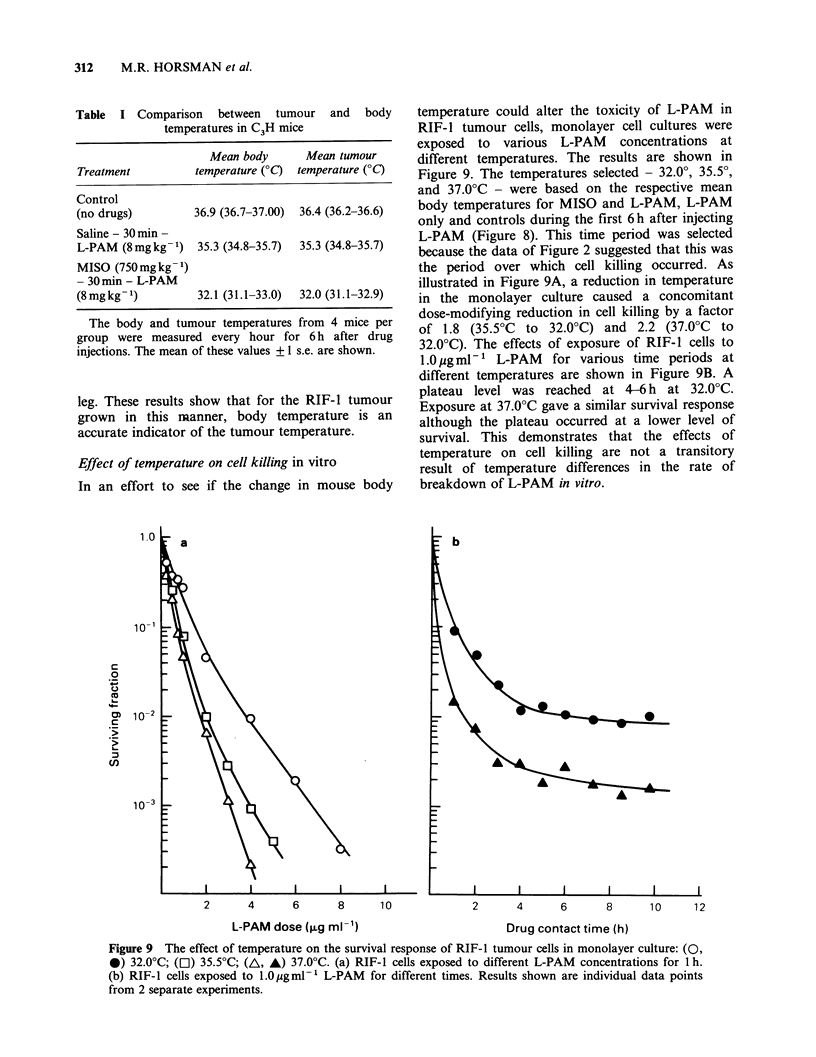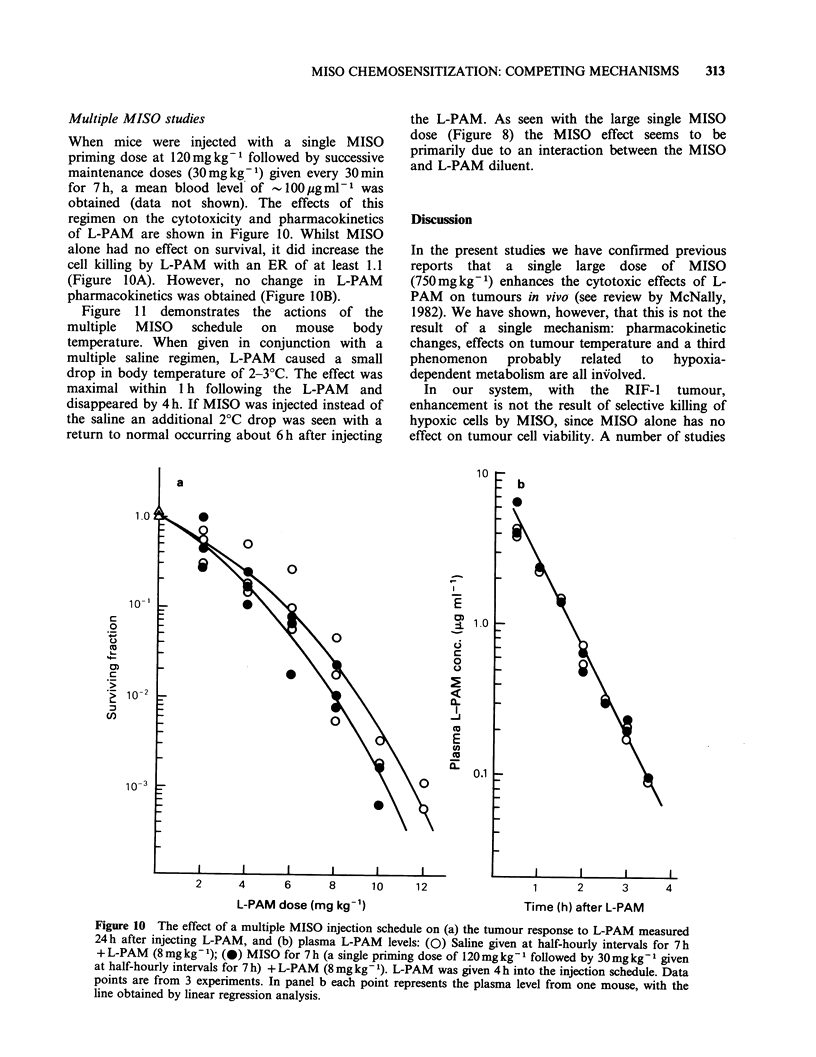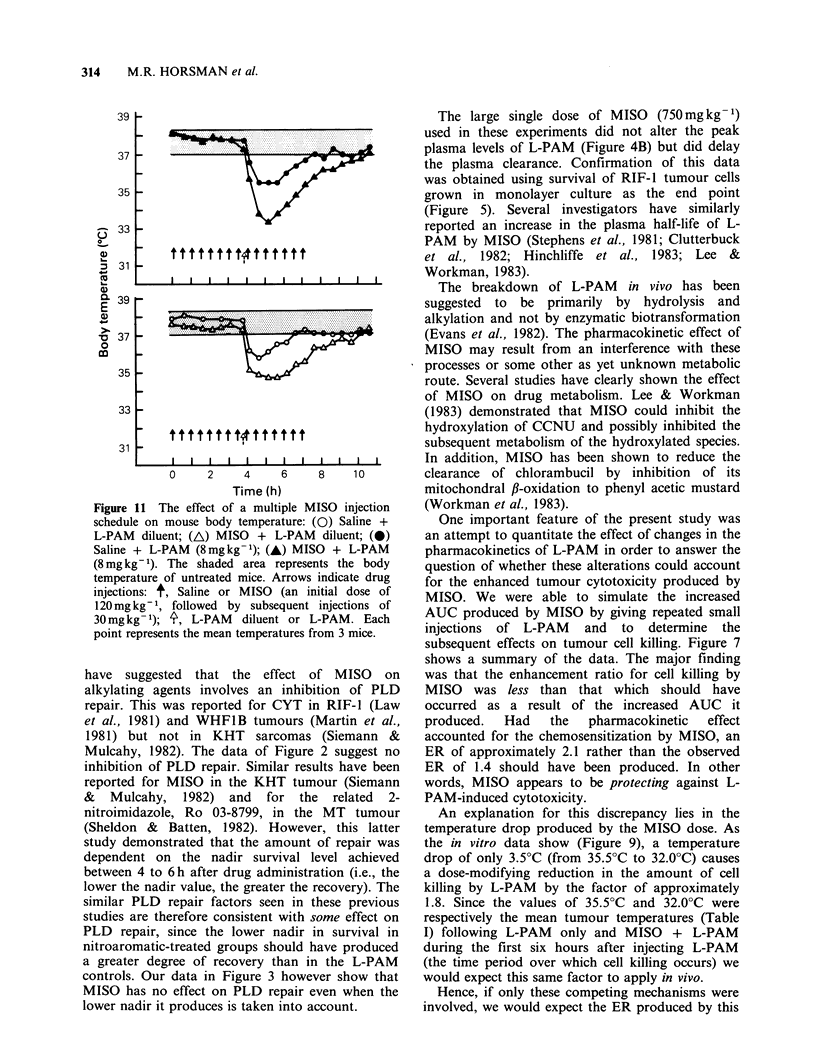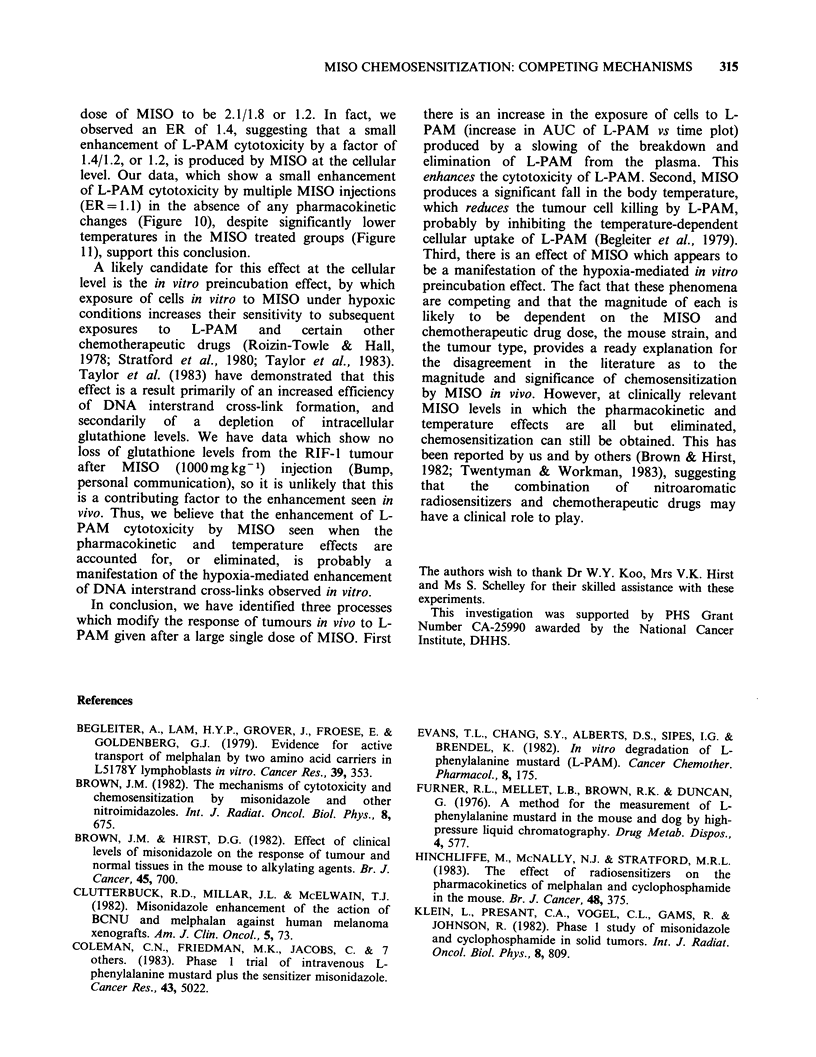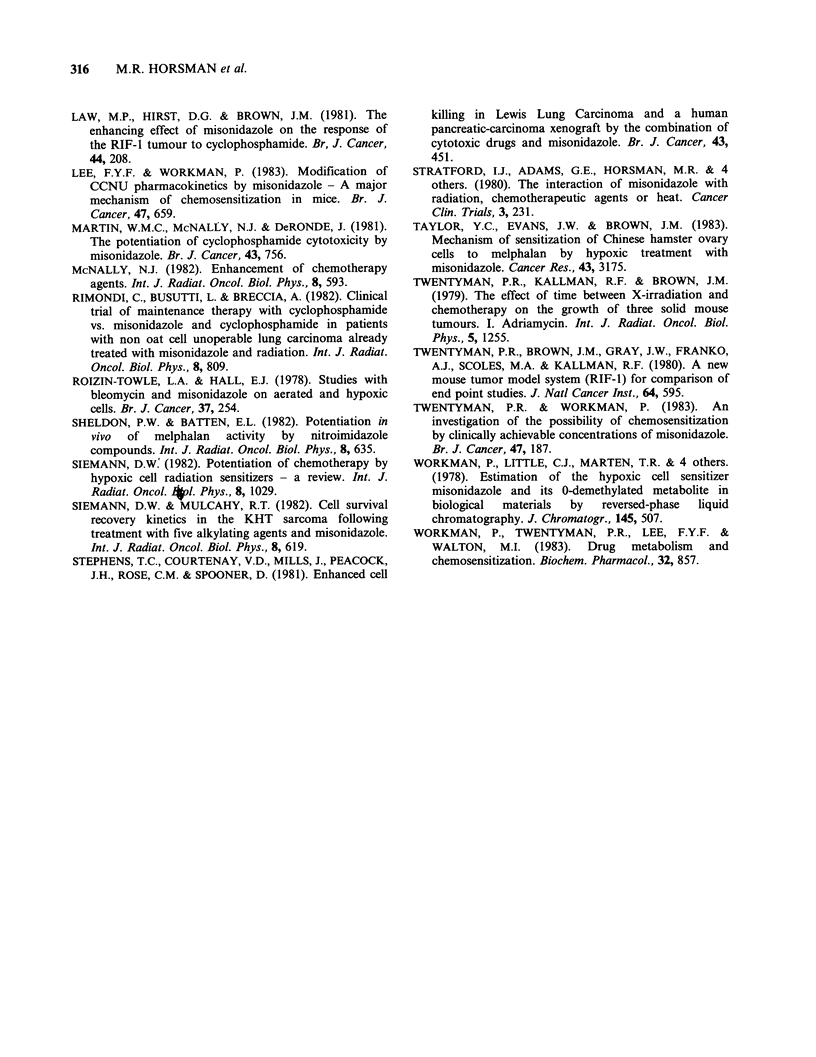# Enhancement of melphalan-induced tumour cell killing by misonidazole: an interaction of competing mechanisms.

**DOI:** 10.1038/bjc.1984.177

**Published:** 1984-09

**Authors:** M. R. Horsman, J. W. Evans, J. M. Brown

## Abstract

In the present studies we have used the RIF-1 tumour in C3H mice to try to identify the mechanism(s) responsible for the enhancement of melphalan (L-PAM) induced tumour cell killing by the 2-nitroimidazole misonidazole (MISO). Most of this work was done with a single large dose of MISO (750 mg kg-1) given 30 min before injection of L-PAM. We found no effect of MISO on the repair of L-PAM-induced potentially lethal damage (PLD) as measured using an in vitro clonogenic survival assay. However, we identified three interrelated and competing processes which affect tumour cell killing by L-PAM subsequent to MISO injection. First, MISO reduces the clearance rate of L-PAM from the blood, an effect which enhances the cell killing by L-PAM. Second, MISO reduces the body temperature which produces a significant reduction in L-PAM cytotoxicity. Third, there is an enhancement of L-PAM cell killing by MISO over and above these two competing processes which is probably a result of the same mechanism by which cells in vitro are sensitized to L-PAM by pre-exposure to MISO under hypoxic conditions.


*Br. J. Cancer* (1984), **50**, 305–316

### Enhancement of melphalan-induced tumour cell killing by misonidazole: An interaction of competing mechanisms

M.R. Horsman, J.W. Evans & J.M. Brown

*Division of Radiobiology Research, Department of Radiology, Stanford University Medical School, Stanford, CA 94305, USA.*

**Summary** In the present studies we have used the RIF-1 tumour in C3H mice to try to identify the mechanism(s) responsible for the enhancement of melphalan (L-PAM) induced tumour cell killing by the 2-nitroimidazole misonidazole (MISO). Most of this work was done with a single large dose of MISO ($750 \, mg \, kg^{-1}$) given 30 min before injection of L-PAM. We found no effect of MISO on the repair of L-PAM-induced potentially lethal damage (PLD) as measured using an *in vitro* clonogenic survival assay. However, we identified three interrelated and competing processes which affect tumour cell killing by L-PAM subsequent to MISO injection. First, MISO reduces the clearance rate of L-PAM from the blood, an effect which *enhances* the cell killing by L-PAM. Second, MISO reduces the body temperature which produces a significant *reduction* in L-PAM cytotoxicity. Third, there is an enhancement of L-PAM cell killing by MISO over and above these two competing processes which is probably a result of the same mechanism by which cells *in vitro* are sensitized to L-PAM by pre-exposure to MISO under hypoxic conditions.

Considerable interest has been shown in combining conventional anti-tumour agents and nitroaromatic radiation sensitizers, especially the 2-nitroimidazoles. Of the chemotherapeutic agents tested in mouse tumour models, the results to date indicate that misonidazole (MISO) shows its greatest interaction with the bifunctional alkylating agents cyclophosphamide (CYT), melphalan (L-PAM), and the nitrosoureas, especially CCNU (1-[2-chloroethyl]-3-cyclohexyl-1-nitrosourea) (McNally, 1982; Siemann, 1982).

In general, tumour toxicity is enhanced to a greater degree than is the response of normal tissues. As a result, Phase I clinical studies of MISO in combination with CYT and L-PAM are currently in progress (Rimondi *et al.*, 1982; Klein *et al.*, 1982; Coleman *et al.*, 1983).

At the present time, the mechanisms for the chemosensitization of alkylating agents by MISO remain unclear. Several processes have been implicated. These include selective killing of hypoxic cells by MISO, changes in the pharmacokinetics and/or metabolism of drugs by MISO, interference with the repair of potentially lethal damage (PLD) induced by the chemotherapeutic agent, and a manifestation of the *in vitro* chemosensitization obtained by pre-exposure of cells to MISO under hypoxic conditions (Brown, 1982; Siemann, 1982).

In the present study we have used the RIF-1 tumour model to investigate the mechanism by which MISO sensitizes these tumour cells to L-PAM.

Correspondence: M.R. Horsman.
Received 1 February 1984; accepted 30 May 1984.

### Materials and methods

### *Tumour system*

The RIF-1 tumour used in the present study is a non-immunogenic sarcoma in its syngeneic host (the C3H/Km mouse) and has been developed for *in vivo–in vitro* assay (Twentyman *et al.*, 1980). It is routinely maintained by passage *in vitro*. Solid tumours were produced in 3–4 month old female C3H/Km mice by innoculating $2 \times 10^5$ cells in a volume of 0.05 ml into the base of the gastrocnemius muscle in the right rear leg. Tumour growth was followed by measuring two leg diameters at right angles and tumour volume was estimated from a calibration curve of tumour weight ($\simeq$tumour volume) plotted as a function of the product of the two leg diameters (Twentyman *et al.*, 1979). Drug treatments were given when the tumours were 300–600 mg.

### *Drug treatments*

All drug solutions were prepared immediately prior to injection. MISO (obtained from the US National Cancer Institute) was dissolved in a sterile saline solution (0.9% NaCl). L-PAM (Burroughs Wellcome Co., Research Triangle Park, NC) was prepared by dissolving 10 mg in 1 ml of 95% ethanol + 5% HCl. It was subsequently diluted to the required concentration in a 60% solution of propylene glycol in saline. In the experiments in which multiple doses of L-PAM were administered, the same drug preparation procedures were used to give a concentration of $0.8 \, mg \, ml^{-1}$. Subsequent dilutions were carried out in saline. Prepared drug concentrations were varied so that a constant

© The Macmillan Press Ltd., 1984

injection volume could be used for each drug. Solutions of MISO ($0.03\,ml\,g^{-1}$ body wt) and L-PAM ($0.01\,ml\,g^{-1}$ body wt) were injected i.p. The L-PAM was kept on ice once prepared. In the multiple MISO studies, mice were injected with a single priming dose of MISO ($120\,mg\,kg^{-1}$) followed by smaller maintenance doses ($30\,mg\,kg^{-1}$) given at half-hourly intervals for 7h. The L-PAM was injected 4h after the start of the experiment and immediately before the 9th MISO maintenance injection.

*Tumour survival assay*

The survival of RIF-1 tumour cells was determined by excising tumours at various times, up to and including 24h, after injecting L-PAM. Three tumours were combined and used for each data point. The tumours were minced by high speed chopping and a single cell suspension prepared by incubating the tumours for 30min at 37°C with an enzyme "cocktail" of 0.05% pronase, 0.02% DNase and 0.015% collagenase in Hank's Balanced salt solution (HBSS). The resulting cell suspensions were filtered through a fine, stainless steel screen ($100\,\mu$ mesh), centrifuged (1500rpm; 10min) and the cell pellet resuspended in Waymouth's medium plus 15% foetal calf serum (Waymouth's + 15% FCS). The density of viable cells was determined by counting, in a haemacytometer, the number of cells which excluded trypan blue. Cells were serially diluted in Waymouth's + 15% FCS and plated into plastic petri dishes at 3 pre-determined dilutions per group. After 13 days incubation at 37°C in an atmosphere of Air + 5% $CO_2$ the dishes were stained with crystal violet and the number of colonies, with at least 50 cells, counted.

Surviving fractions were calculated as the number of colonies counted, divided by the number of cells plated, corrected for plating efficiency (survival of untreated cells, which typically fell in the range 20–40%).

*Bioassay*

The possible interaction between L-PAM and MISO in the blood and its subsequent effect on survival of RIF-1 tumour cells was investigated by exposing RIF-1 cells in monolayer to plasma from drug treated animals. C3H mice were injected with either saline or MISO and L-PAM in the usual way. At various times after the L-PAM injection the mice were bled by cardiac puncture under diethyl ether anaesthesia. The blood from 4–5 animals was combined, and the plasma obtained by centrifugation (3000rpm; 5min) of heparinized whole blood. The plasma was diluted by 50% with serum from mice injected with only the drug solvents and 2.0ml was then transferred to 60mm petri-dishes containing RIF-1 tumour cells. These cells had been plated at a concentration of $1\times10^6$ cells/dish, at least 3h before exposure to the plasma, to allow them sufficient time to attach to the plastic surface. After incubating with the plasma for 2h (37°C; Air + 5% $CO_2$) the plates were rinsed once with sterile saline, exposed to 0.05% trypsin (10min at 37°C) and survival of the subsequent single cell suspension assayed as outlined earlier.

In two experiments the MISO was not injected into the L-PAM treated mice. In the first instance it was given to mice which were injected with only the L-PAM solvent and the plasma from both MISO and L-PAM treated mice mixed immediately before addition to RIF-1 tumour cells in culture. Secondly, the MISO was not metabolized by the mice at all. Instead it was prepared in saline, diluted with untreated mouse plasma and mixed with L-PAM treated plasma just before exposure to the RIF-1 cells. In all conditions the total volume of plasma added to the plates and the volume of L-PAM containing plasma remained constant.

*Measurement of plasma drug levels*

Plasma was obtained as described earlier and stored at −20°C. Drug concentrations were analysed by reversed phase high-performance liquid chromatography (HPLC).

*L-PAM concentration* L-PAM blood concentrations were determined by the procedure described by Furner *et al.* (1976). The plasma sample was diluted (9:1 v/v) with Dansyl proline ($150\,\mu g\,ml^{-1}$ in Methanol) which served as an internal standard. This solution was mixed in the ratio of 11:9 (v/v) with a deproteinizing agent (10% perchloric acid in methanol + 0.1% acetic acid). The resulting solution was vortexed for 15sec and centrifuged (3000rpm; 8min). The supernatant was removed and mixed with $KH_2PO_4$ (2:1; v/v) to precipitate the perchlorate. Following a further vortexing and centrifugation the supernatant was removed and passed through a BONDAPAK $C_{18}$ column (3.9mm × 30cm). The eluent consisted of 52% methanol + 1% acetic acid. Drug levels were measured with a UV detector (Model 450 variable wavelength detector) at 254nm. Results were collected on a data module chart recorder. Quantitation of drug concentration was by peak area with reference to linear calibration curves. The columns, detector and chart recorder were all purchased from Waters Associates, Milford, Mass. Throughout the preparation procedure all solutions were kept at 4°C.

*MISO concentration* The method used to determine MISO blood levels was based on the procedure described by Workman *et al.* (1978). Essentially the plasma was initially diluted (1:1, v/v) with an internal standard. In this instance this was the compound Ro 07-0741 (1-(2-nitroimidazol-1-yl)-3-fluropropan-2-ol; 10 $\mu$g ml$^{-1}$ in methanol). Samples were then vortexed for 15 sec, centrifuged (3000 rpm; 10 min) and the resulting supernatant run on a RCM-100 (Radial Compression Module) with a Radial-PAK $C_{18}$ cartridge (10 $\mu$, 8 mm × 10 cm). The eluent was 30% methanol and the drug levels were measured at 324 nm. Drug concentrations were determined as described above.

### In vitro *temperature studies*

The effect of temperature on L-PAM toxicity was investigated by exposing RIF-1 tumour cells in monolayer culture to various L-PAM concentrations at either 37.0°C, 35.5°C, or 32.0°C. Exponentially growing tumour cells were plated in full growth medium at different cell concentrations (between $10^2$ to $10^5$ cells/petri dish) depending on the expected survival level, and incubated at 37°C in an atmosphere of Air + 5% $CO_2$ for 2 h to allow the cells time to attach to the plastic surface. These cells were then incubated for one additional hour at the temperature at which the drug exposures would be carried out. After this time the medium was removed by aspiration, 2.5 ml of the appropriate drug concentrations added and the plates returned to the required temperature. At various times the drug solutions were removed, the plates washed once with sterile saline and fresh media added. All the plates were returned to the 37°C incubator for 13 days before assaying for colony formation in the usual way.

### *Temperature measurements*

Body temperatures were determined using a rectally inserted thermocouple (Baily Instruments, Saddle Brooke, NJ 07662). Tumour temperatures were measured by inserting a microprobe thermocouple (Baily Instruments) into the tumour of restrained unanaesthetised mice.

### *Analysis of data*

For all survival results, individual data points from at least 3 experiments are shown. The curves are the "best-fit" by eye to the data. Where enhancement ratios (ER's) are quoted, these are the average values from the survival curves down to a surviving fraction of $10^{-3}$. The ER is defined as the ratio of drug concentration for L-PAM alone to that obtained with L-PAM and MISO at the same survival level. The lines of "best-fit" for the pharmacokinetic data were determined by linear regression analysis in the regions where exponential decays operated. Drug half-lives (with 95% confidence limits) were calculated from these lines.

### Results

### *Tumour cell survival studies*

Mice bearing RIF-1 tumours were injected with MISO (750 mg kg$^{-1}$) or saline 30 min before various single doses of L-PAM. Tumours were excised 24 h after the L-PAM injection and cell survival assayed. The results are shown in Figure 1. Increasing doses of L-PAM caused increasing amounts of cell kill. MISO alone at the dose used had no effect on survival but it did enhance the cytotoxicity of L-PAM. Although there was some variability between experiments so that overlap in the data points was seen, the MISO + L-PAM treatment groups were always lower than the L-PAM-only groups within each experiment. The data shown in Figure 1 suggest that MISO had a dose modifying effect for all L-PAM doses, giving a mean enhancement ratio (ER) of 1.4.

**Figure 1** The effect of MISO on the response of the RIF-1 tumour to L-PAM as measured by tumour survival, assayed 24 h after drug administration: (△) MISO (750 mg kg$^{-1}$) – 30 min – L-PAM diluent; (○) Saline – 30 min – L-PAM; (●) MISO (750 mg kg$^{-1}$) – 30 min – L-PAM. Individual data points from 4 separate experiments are shown.

Figure 2 shows the effect on survival of varying the time of removal of tumours from the animals following an L-PAM injection. When mice were given saline 30 min before a single injection of L-PAM (8 mg kg$^{-1}$) significant toxicity was seen even when the tumours were removed within 2 h following the L-PAM dose. Survival continued to decrease with a nadir being reached at 4–6 h. This was followed by repair of L-PAM induced potentially lethal damage (PLD), shown by an increasing surviving fraction. MISO (750 mg kg$^{-1}$) alone had little or no effect on tumour cell survival over the 24 h period of study. However, it potentiated the effect of L-PAM. The degree of enhancement increased with time, with the maximum effect being observed at 6 h. No further increase in killing was seen and for the remaining time period the L-PAM alone and the L-PAM with MISO curves were parallel, suggesting little or no effect on the repair of PLD.

In order to investigate this further, curves similar to those shown in Figure 2 were produced for 5 different L-PAM concentrations (2, 4, 6, 8, and 10 mg kg$^{-1}$) with or without MISO (750 mg kg$^{-1}$). From these data the lowest survival level attained with each L-PAM concentration was determined, regardless of the time at which it occurred. Generally, however, the results were similar to those in Figure 2, with the nadir being reached at 4–6 h. These values are plotted in Figure 3, as a function of the difference between the nadir level and survival at 24 h (an indication of the total amount of PLD repair occurring). Although in every instance a greater amount of cell killing was achieved when MISO was given with L-PAM than for L-PAM alone, no difference is seen between the L-PAM data and the L-PAM and MISO values. This confirms that MISO has no effect on L-PAM-induced PLD repair in the RIF-1 tumour.

**Figure 2** Survival of RIF-1 tumour cells as a function of time after drug administration: (△) MISO (750 mg kg$^{-1}$) – 30 min – L-PAM diluent; (○) Saline – 30 min – L-PAM (8 mg kg$^{-1}$); (●) MISO (750 mg kg$^{-1}$) – 30 min – L-PAM (8 mg kg$^{-1}$). The results from 3 experiments are shown.

**Figure 3** Relationship between the maximum amount of cell killing obtained and the degree of repair. Curves similar to those shown in **Figure 2** were produced for different L-PAM concentrations ± MISO and both the lowest level of survival reached and the degree of recovery (repair factor) were determined: (○) Saline – 30 min – L-PAM; (●) MISO (750 mg kg$^{-1}$) – 30 min – L-PAM. Data from 3 different experiments are shown.

*Pharmacokinetic studies*

Figure 4 shows plasma levels for both MISO and L-PAM as a function of time following injections

**Figure 4** Plasma levels of MISO (a) and L-PAM (b) in tumour-bearing C3H mice as a function of time after L-PAM administration: (△) MISO (750 mg $kg^{-1}$) – 30 min – L-PAM diluent; (○) Saline – 30 min – L-PAM (8 mg $kg^{-1}$); (●, ▲) MISO (750 mg $kg^{-1}$) – 30 min – L-PAM (8 mg $kg^{-1}$). Each point represents the plasma level from one mouse, with the results of 3 separate experiments shown.

of either saline or MISO (750 mg $kg^{-1}$) 30 min before L-PAM (8 mg $kg^{-1}$). A peak level for MISO occurs 30 min after an injection of L-PAM or L-PAM solvent (Figure 4A). This is followed by a gradual decline with an elimination half-life of ~5 h. The presence of L-PAM does not alter the peak MISO level or the clearance rate. However, MISO (750 mg $kg^{-1}$) given 30 min before a dose of 8 mg $kg^{-1}$ L-PAM slowed the rate of clearance of L-PAM from the blood (Figure 4B). The elimination half-life of L-PAM was extended from 38.3 min (32.7–46.1) to 86.6 min (75.3–101.7). This dose of MISO had no effect on the peak plasma level of L-PAM.

The effect of MISO on L-PAM pharmacokinetics was also studied using survival of RIF-tumour cells grown in monolayer culture as the end point. The results are shown in Figure 5. Plasma was removed at various times from mice given either saline or MISO (750 mg $kg^{-1}$) 30 min before L-PAM (8 mg $kg^{-1}$). Following dilution (1:1) with plasma from mice given only the drug solvents it was transferred to the RIF-1 cells in culture. Figure 5 shows that with a 2 h exposure to the plasma, significant cell killing was seen as early as 30 min after injecting the L-PAM. Cell killing decreased as time in the mouse increased, with the cytotoxicity being lost by 4 h. When MISO was given 30 min before L-PAM an enhancement of the L-PAM induced killing was obtained. These data correlate well with the result in Figure 4B.

In order to test for any possible interaction at the cellular level, plasma from mice receiving MISO alone was therefore combined with plasma from mice receiving L-PAM alone and added to the RIF-1 cells *in vitro*. As shown in Figure 5, there was no effect of mixing the drugs after metabolism in the mice. Similar results are seen even if the MISO was not metabolized in the mice, but instead prepared and added to the L-PAM directly. In this experiment the MISO concentration added was equivalent to the concentrations in the blood at the various times of assay (values taken from Figure 4A).

### *Relationship between pharmacokinetic changes and increased cytotoxicity*

Figure 4B shows that a large single dose of MISO (750 mg $kg^{-1}$) increases the elimination half-life of L-PAM (8 mg $kg^{-1}$). In order to see if this change in L-PAM pharmacokinetics was responsible for the enhanced cytotoxicity produced by MISO, the increased half-life was duplicated by giving mice the same initial dose of L-PAM (8 mg $kg^{-1}$) followed by subsequent smaller L-PAM doses at hourly intervals for 6 h (see legend of Figure 6 for values). Identical groups of mice were given the same priming dose of L-PAM but followed by doses above or below that simulating the pharmacokinetic changes produced by MISO. The pharmacokinetic data on a linear plot are shown in Figure 6. These

**Figure 5** Survival of RIF-1 tumour cells in monolayer culture after a 2 h exposure to plasma from tumour-bearing C3H mice receiving the following: (▼) no drugs; (○) Saline – 30 min – L-PAM (8 mg kg$^{-1}$); (●) MISO (750 mg kg$^{-1}$) – 30 min – L-PAM (8 mg kg$^{-1}$); (□) MISO (750 mg kg$^{-1}$); (△) MISO (750 mg kg$^{-1}$) or L-PAM (8 mg kg$^{-1}$) – plasma combined before addition to cells in culture; (▲) L-PAM (8 mg kg$^{-1}$) only – plasma mixed with unmetabolized MISO before exposure to cells; (■) no drugs – plasma combined with unmetabolized MISO before addition to cells in culture. Three separate experiments are shown with each point representing the survival of cells from a single plate having received diluted serum pooled from 4–5 mice.

**Figure 6** Curves of plasma L-PAM concentration as a function of time after injection. Panel (a) shows the mean curves from 4 different experiments for L-PAM only (8 mg kg$^{-1}$, ○) and L-PAM preceded by MISO (750 mg kg$^{-1}$, ●). Panels (b) and (c) show the mean plasma concentration versus time curves for mice given the same initial L-PAM dose (8 mg kg$^{-1}$) followed hourly for 6 h with smaller L-PAM doses. These L-PAM doses are designated X/2 (□); X (△); 3/2X (◇); and 2X (▽), where X is the attempt to simulate the MISO + L-PAM curve in panel a (shown by the dashed line in panels b and c), and was obtained by injecting 2.5, 1.3, 0.7, 0.7, 0.7 and 0.56 mg kg$^{-1}$ at 1, 2, 3, 4, 5 and 6 h respectively. The pooled data from 2 to 3 separate experiments are shown, with each point representing the mean plasma level from one mouse. Lines are the "best-fit" to the data by eye.

results are the mean values for 3 to 4 separate experiments. For each individual experiment the area under the curve (AUC) was determined by weighing the cut-out area of graph paper. Taking the AUC for the single dose of L-PAM (8 mg kg$^{-1}$) as 1.0, we determined the factor by which the AUC for each treatment schedule was increased. Tumour survival, assayed 24 h after the L-PAM priming dose for each injection regimen, was also measured in each experiment and the ER for cell survival calculated from the L-PAM dose-response curve (Figure 1). The results for both the increase in the AUC and the enhancement of tumour cell killing following a single dose of L-PAM, by either the multiple L-PAM dose regimen or the large single injection of MISO 30 min before L-PAM, are shown in Figure 7. Increasing the AUC by either method increased cell killing. However, the enhancement in tumour cell kill for a given increase in AUC was greater for the multiple L-PAM groups than for the MISO and L-PAM groups (ERs of 2.1 and 1.4, respectively, for the same AUC increase).

**Figure 7** Relationship between the change in L-PAM pharmacokinetics and increased tumour cytotoxicity: closed symbols, MISO (750 mg kg$^{-1}$) – 30 min – L-PAM (8 mg kg$^{-1}$); open symbols, Saline – 30 min – L-PAM (8 mg kg$^{-1}$), followed by various smaller L-PAM doses at hourly intervals for 6 h as explained in **Figure 6**: (○) saline; (□) X/2; (△) X; (◇) 3/2X; (▽) 2X. The factor increase in AUC is the increase in area of the data shown in **Figure 6**, taking the AUC for the single dose of L-PAM as 1.0. Enhancement of survival was measured by an excision assay performed 24 h after drug administration. The data points are from 2 to 4 separate experiments with the lines showing the "best-fit" to the data by eye.

*Changes in tumour and body temperature*

A large single dose of L-PAM (8 mg kg$^{-1}$) caused a drop in rectal temperature of about 4°C in C3H

**Figure 8** Mouse body temperature as a function of time after drug administration: (○) Saline – 30 min – L-PAM diluent; (□) MISO (750 mg kg$^{-1}$); (△) MISO (750 mg kg$^{-1}$) – 30 min – L-PAM diluent; (●) Saline – 30 min – L-PAM (8 mg kg$^{-1}$); (▲) MISO (750 mg kg$^{-1}$) – 30 min – L-PAM (8 mg kg$^{-1}$). The shaded area represents the body temperature of untreated mice. Arrows indicate drug injections: ↑, saline or MISO; ⇡, L-PAM diluent or L-PAM. Each point represents the mean temperature from three mice.

mice (Figure 8). This lowered temperature was reached within one hour after the injection of the L-PAM, but was followed by recovery such that 8 h later the body temperature had returned to normal. Most of this effect was due to the L-PAM diluent (Figure 8). The injection of MISO (750 mg kg$^{-1}$) alone produced a slightly greater fall in body temperature than the L-PAM diluent. However, when MISO preceded L-PAM, the fall in body temperature was greater (6–7°C) and more prolonged: no significant recovery was observed, even 8 h after giving the drugs.

Table I shows a comparison between tumour and body temperatures measured in the same mice. Temperature recordings were carried out every hour for 6 h after injecting the drugs and the mean values calculated. No significant difference can be seen between the body and tumour results. This is probably a consequence of the tumours being in a highly vascularized intramuscular site in the mouse

**Table I** Comparison between tumour and body temperatures in $C_3H$ mice

| *Treatment* | *Mean body temperature (°C)* | *Mean tumour temperature (°C)* |
|---|---|---|
| Control (no drugs) | 36.9 (36.7–37.00) | 36.4 (36.2–36.6) |
| Saline – 30 min – L-PAM (8 mg kg$^{-1}$) | 35.3 (34.8–35.7) | 35.3 (34.8–35.7) |
| MISO (750 mg kg$^{-1}$) – 30 min – L-PAM (8 mg kg$^{-1}$) | 32.1 (31.1–33.0) | 32.0 (31.1–32.9) |

The body and tumour temperatures from 4 mice per group were measured every hour for 6 h after drug injections. The mean of these values ±1 s.e. are shown.

leg. These results show that for the RIF-1 tumour grown in this manner, body temperature is an accurate indicator of the tumour temperature.

*Effect of temperature on cell killing* in vitro

In an effort to see if the change in mouse body temperature could alter the toxicity of L-PAM in RIF-1 tumour cells, monolayer cell cultures were exposed to various L-PAM concentrations at different temperatures. The results are shown in Figure 9. The temperatures selected – 32.0°, 35.5°, and 37.0°C – were based on the respective mean body temperatures for MISO and L-PAM, L-PAM only and controls during the first 6 h after injecting L-PAM (Figure 8). This time period was selected because the data of Figure 2 suggested that this was the period over which cell killing occurred. As illustrated in Figure 9A, a reduction in temperature in the monolayer culture caused a concomitant dose-modifying reduction in cell killing by a factor of 1.8 (35.5°C to 32.0°C) and 2.2 (37.0°C to 32.0°C). The effects of exposure of RIF-1 cells to 1.0 μg ml$^{-1}$ L-PAM for various time periods at different temperatures are shown in Figure 9B. A plateau level was reached at 4–6 h at 32.0°C. Exposure at 37.0°C gave a similar survival response although the plateau occurred at a lower level of survival. This demonstrates that the effects of temperature on cell killing are not a transitory result of temperature differences in the rate of breakdown of L-PAM *in vitro*.

**Figure 9** The effect of temperature on the survival response of RIF-1 tumour cells in monolayer culture: (○, ●) 32.0°C; (□) 35.5°C; (△, ▲) 37.0°C. (a) RIF-1 cells exposed to different L-PAM concentrations for 1 h. (b) RIF-1 cells exposed to 1.0 μg ml$^{-1}$ L-PAM for different times. Results shown are individual data points from 2 separate experiments.

*Multiple MISO studies*

When mice were injected with a single MISO priming dose at $120 mg kg^{-1}$ followed by successive maintenance doses ($30 mg kg^{-1}$) given every 30 min for 7 h, a mean blood level of $\sim 100 \mu g ml^{-1}$ was obtained (data not shown). The effects of this regimen on the cytotoxicity and pharmacokinetics of L-PAM are shown in Figure 10. Whilst MISO alone had no effect on survival, it did increase the cell killing by L-PAM with an ER of at least 1.1 (Figure 10A). However, no change in L-PAM pharmacokinetics was obtained (Figure 10B).

Figure 11 demonstrates the actions of the multiple MISO schedule on mouse body temperature. When given in conjunction with a multiple saline regimen, L-PAM caused a small drop in body temperature of 2–3°C. The effect was maximal within 1 h following the L-PAM and disappeared by 4 h. If MISO was injected instead of the saline an additional 2°C drop was seen with a return to normal occurring about 6 h after injecting the L-PAM. As seen with the large single MISO dose (Figure 8) the MISO effect seems to be primarily due to an interaction between the MISO and L-PAM diluent.

### Discussion

In the present studies we have confirmed previous reports that a single large dose of MISO ($750 mg kg^{-1}$) enhances the cytotoxic effects of L-PAM on tumours *in vivo* (see review by McNally, 1982). We have shown, however, that this is not the result of a single mechanism: pharmacokinetic changes, effects on tumour temperature and a third phenomenon probably related to hypoxia-dependent metabolism are all involved.

In our system, with the RIF-1 tumour, enhancement is not the result of selective killing of hypoxic cells by MISO, since MISO alone has no effect on tumour cell viability. A number of studies

**Figure 10** The effect of a multiple MISO injection schedule on (a) the tumour response to L-PAM measured 24 h after injecting L-PAM, and (b) plasma L-PAM levels: (○) Saline given at half-hourly intervals for 7 h + L-PAM ($8 mg kg^{-1}$); (●) MISO for 7 h (a single priming dose of $120 mg kg^{-1}$ followed by $30 mg kg^{-1}$ given at half-hourly intervals for 7 h) + L-PAM ($8 mg kg^{-1}$). L-PAM was given 4 h into the injection schedule. Data points are from 3 experiments. In panel b each point represents the plasma level from one mouse, with the line obtained by linear regression analysis.

**Figure 11** The effect of a multiple MISO injection schedule on mouse body temperature: (○) Saline + L-PAM diluent; (△) MISO + L-PAM diluent; (●) Saline + L-PAM (8 mg kg$^{-1}$); (▲) MISO + L-PAM (8 mg kg$^{-1}$). The shaded area represents the body temperature of untreated mice. Arrows indicate drug injections: ↑, Saline or MISO (an initial dose of 120 mg kg$^{-1}$, followed by subsequent injections of 30 mg kg$^{-1}$); ⇡, L-PAM diluent or L-PAM. Each point represents the mean temperatures from 3 mice.

have suggested that the effect of MISO on alkylating agents involves an inhibition of PLD repair. This was reported for CYT in RIF-1 (Law *et al.*, 1981) and WHF1B tumours (Martin *et al.*, 1981) but not in KHT sarcomas (Siemann & Mulcahy, 1982). The data of Figure 2 suggest no inhibition of PLD repair. Similar results have been reported for MISO in the KHT tumour (Siemann & Mulcahy, 1982) and for the related 2-nitroimidazole, Ro 03-8799, in the MT tumour (Sheldon & Batten, 1982). However, this latter study demonstrated that the amount of repair was dependent on the nadir survival level achieved between 4 to 6 h after drug administration (i.e., the lower the nadir value, the greater the recovery). The similar PLD repair factors seen in these previous studies are therefore consistent with *some* effect on PLD repair, since the lower nadir in survival in nitroaromatic-treated groups should have produced a greater degree of recovery than in the L-PAM controls. Our data in Figure 3 however show that MISO has no effect on PLD repair even when the lower nadir it produces is taken into account.

The large single dose of MISO (750 mg kg$^{-1}$) used in these experiments did not alter the peak plasma levels of L-PAM (Figure 4B) but did delay the plasma clearance. Confirmation of this data was obtained using survival of RIF-1 tumour cells grown in monolayer culture as the end point (Figure 5). Several investigators have similarly reported an increase in the plasma half-life of L-PAM by MISO (Stephens *et al.*, 1981; Clutterbuck *et al.*, 1982; Hinchliffe *et al.*, 1983; Lee & Workman, 1983).

The breakdown of L-PAM *in vivo* has been suggested to be primarily by hydrolysis and alkylation and not by enzymatic biotransformation (Evans *et al.*, 1982). The pharmacokinetic effect of MISO may result from an interference with these processes or some other as yet unknown metabolic route. Several studies have clearly shown the effect of MISO on drug metabolism. Lee & Workman (1983) demonstrated that MISO could inhibit the hydroxylation of CCNU and possibly inhibited the subsequent metabolism of the hydroxylated species. In addition, MISO has been shown to reduce the clearance of chlorambucil by inhibition of its mitochondral $\beta$-oxidation to phenyl acetic mustard (Workman *et al.*, 1983).

One important feature of the present study was an attempt to quantitate the effect of changes in the pharmacokinetics of L-PAM in order to answer the question of whether these alterations could account for the enhanced tumour cytotoxicity produced by MISO. We were able to simulate the increased AUC produced by MISO by giving repeated small injections of L-PAM and to determine the subsequent effects on tumour cell killing. Figure 7 shows a summary of the data. The major finding was that the enhancement ratio for cell killing by MISO was *less* than that which should have occurred as a result of the increased AUC it produced. Had the pharmacokinetic effect accounted for the chemosensitization by MISO, an ER of approximately 2.1 rather than the observed ER of 1.4 should have been produced. In other words, MISO appears to be *protecting* against L-PAM-induced cytotoxicity.

An explanation for this discrepancy lies in the temperature drop produced by the MISO dose. As the *in vitro* data show (Figure 9), a temperature drop of only 3.5°C (from 35.5°C to 32.0°C) causes a dose-modifying reduction in the amount of cell killing by L-PAM by the factor of approximately 1.8. Since the values of 35.5°C and 32.0°C were respectively the mean tumour temperatures (Table I) following L-PAM only and MISO + L-PAM during the first six hours after injecting L-PAM (the time period over which cell killing occurs) we would expect this same factor to apply *in vivo*.

Hence, if only these competing mechanisms were involved, we would expect the ER produced by this dose of MISO to be 2.1/1.8 or 1.2. In fact, we observed an ER of 1.4, suggesting that a small enhancement of L-PAM cytotoxicity by a factor of 1.4/1.2, or 1.2, is produced by MISO at the cellular level. Our data, which show a small enhancement of L-PAM cytotoxicity by multiple MISO injections (ER = 1.1) in the absence of any pharmacokinetic changes (Figure 10), despite significantly lower temperatures in the MISO treated groups (Figure 11), support this conclusion.

A likely candidate for this effect at the cellular level is the *in vitro* preincubation effect, by which exposure of cells *in vitro* to MISO under hypoxic conditions increases their sensitivity to subsequent exposures to L-PAM and certain other chemotherapeutic drugs (Roizin-Towle & Hall, 1978; Stratford *et al.*, 1980; Taylor *et al.*, 1983). Taylor *et al.* (1983) have demonstrated that this effect is a result primarily of an increased efficiency of DNA interstrand cross-link formation, and secondarily of a depletion of intracellular glutathione levels. We have data which show no loss of glutathione levels from the RIF-1 tumour after MISO ($1000\,mg\,kg^{-1}$) injection (Bump, personal communication), so it is unlikely that this is a contributing factor to the enhancement seen *in vivo*. Thus, we believe that the enhancement of L-PAM cytotoxicity by MISO seen when the pharmacokinetic and temperature effects are accounted for, or eliminated, is probably a manifestation of the hypoxia-mediated enhancement of DNA interstrand cross-links observed *in vitro*.

In conclusion, we have identified three processes which modify the response of tumours *in vivo* to L-PAM given after a large single dose of MISO. First there is an increase in the exposure of cells to L-PAM (increase in AUC of L-PAM *vs* time plot) produced by a slowing of the breakdown and elimination of L-PAM from the plasma. This *enhances* the cytotoxicity of L-PAM. Second, MISO produces a significant fall in the body temperature, which *reduces* the tumour cell killing by L-PAM, probably by inhibiting the temperature-dependent cellular uptake of L-PAM (Begleiter *et al.*, 1979). Third, there is an effect of MISO which appears to be a manifestation of the hypoxia-mediated *in vitro* preincubation effect. The fact that these phenomena are competing and that the magnitude of each is likely to be dependent on the MISO and chemotherapeutic drug dose, the mouse strain, and the tumour type, provides a ready explanation for the disagreement in the literature as to the magnitude and significance of chemosensitization by MISO *in vivo*. However, at clinically relevant MISO levels in which the pharmacokinetic and temperature effects are all but eliminated, chemosensitization can still be obtained. This has been reported by us and by others (Brown & Hirst, 1982; Twentyman & Workman, 1983), suggesting that the combination of nitroaromatic radiosensitizers and chemotherapeutic drugs may have a clinical role to play.

The authors wish to thank Dr W.Y. Koo, Mrs V.K. Hirst and Ms S. Schelley for their skilled assistance with these experiments.

This investigation was supported by PHS Grant Number CA-25990 awarded by the National Cancer Institute, DHHS.

### References

BEGLEITER, A., LAM, H.Y.P., GROVER, J., FROESE, E. & GOLDENBERG, G.J. (1979). Evidence for active transport of melphalan by two amino acid carriers in L5178Y lymphoblasts *in vitro*. *Cancer Res.*, **39**, 353.

BROWN, J.M. (1982). The mechanisms of cytotoxicity and chemosensitization by misonidazole and other nitroimidazoles. *Int. J. Radiat. Oncol. Biol. Phys.*, **8**, 675.

BROWN, J.M. & HIRST, D.G. (1982). Effect of clinical levels of misonidazole on the response of tumour and normal tissues in the mouse to alkylating agents. *Br. J. Cancer*, **45**, 700.

CLUTTERBUCK, R.D., MILLAR, J.L. & MCELWAIN, T.J. (1982). Misonidazole enhancement of the action of BCNU and melphalan against human melanoma xenografts. *Am. J. Clin. Oncol.*, **5**, 73.

COLEMAN, C.N., FRIEDMAN, M.K., JACOBS, C. & 7 others. (1983). Phase 1 trial of intravenous L-phenylalanine mustard plus the sensitizer misonidazole. *Cancer Res.*, **43**, 5022.

EVANS, T.L., CHANG, S.Y., ALBERTS, D.S., SIPES, I.G. & BRENDEL, K. (1982). *In vitro* degradation of L-phenylalanine mustard (L-PAM). *Cancer Chemother. Pharmacol.*, **8**, 175.

FURNER, R.L., MELLET, L.B., BROWN, R.K. & DUNCAN, G. (1976). A method for the measurement of L-phenylalanine mustard in the mouse and dog by high-pressure liquid chromatography. *Drug Metab. Dispos.*, **4**, 577.

HINCHLIFFE, M., MCNALLY, N.J. & STRATFORD, M.R.L. (1983). The effect of radiosensitizers on the pharmacokinetics of melphalan and cyclophosphamide in the mouse. *Br. J. Cancer*, **48**, 375.

KLEIN, L., PRESANT, C.A., VOGEL, C.L., GAMS, R. & JOHNSON, R. (1982). Phase 1 study of misonidazole and cyclophosphamide in solid tumors. *Int. J. Radiat. Oncol. Biol. Phys.*, **8**, 809.

LAW, M.P., HIRST, D.G. & BROWN, J.M. (1981). The enhancing effect of misonidazole on the response of the RIF-1 tumour to cyclophosphamide. *Br, J. Cancer,* **44,** 208.

LEE, F.Y.F. & WORKMAN, P. (1983). Modification of CCNU pharmacokinetics by misonidazole – A major mechanism of chemosensitization in mice. *Br. J. Cancer,* **47,** 659.

MARTIN, W.M.C., McNALLY, N.J. & DeRONDE, J. (1981). The potentiation of cyclophosphamide cytotoxicity by misonidazole. *Br. J. Cancer,* **43,** 756.

McNALLY, N.J. (1982). Enhancement of chemotherapy agents. *Int. J. Radiat. Oncol. Biol. Phys.,* **8,** 593.

RIMONDI, C., BUSUTTI, L. & BRECCIA, A. (1982). Clinical trial of maintenance therapy with cyclophosphamide vs. misonidazole and cyclophosphamide in patients with non oat cell unoperable lung carcinoma already treated with misonidazole and radiation. *Int. J. Radiat. Oncol. Biol. Phys.,* **8,** 809.

ROIZIN-TOWLE, L.A. & HALL, E.J. (1978). Studies with bleomycin and misonidazole on aerated and hypoxic cells. *Br. J. Cancer,* **37,** 254.

SHELDON, P.W. & BATTEN, E.L. (1982). Potentiation *in vivo* of melphalan activity by nitroimidazole compounds. *Int. J. Radiat. Oncol. Biol. Phys.,* **8,** 635.

SIEMANN, D.W. (1982). Potentiation of chemotherapy by hypoxic cell radiation sensitizers – a review. *Int. J. Radiat. Oncol. Biol. Phys.,* **8,** 1029.

SIEMANN, D.W. & MULCAHY, R.T. (1982). Cell survival recovery kinetics in the KHT sarcoma following treatment with five alkylating agents and misonidazole. *Int. J. Radiat. Oncol. Biol. Phys.,* **8,** 619.

STEPHENS, T.C., COURTENAY, V.D., MILLS, J., PEACOCK, J.H., ROSE, C.M. & SPOONER, D. (1981). Enhanced cell killing in Lewis Lung Carcinoma and a human pancreatic-carcinoma xenograft by the combination of cytotoxic drugs and misonidazole. *Br. J. Cancer,* **43,** 451.

STRATFORD, I.J., ADAMS, G.E., HORSMAN, M.R. & 4 others. (1980). The interaction of misonidazole with radiation, chemotherapeutic agents or heat. *Cancer Clin. Trials,* **3,** 231.

TAYLOR, Y.C., EVANS, J.W. & BROWN, J.M. (1983). Mechanism of sensitization of Chinese hamster ovary cells to melphalan by hypoxic treatment with misonidazole. *Cancer Res.,* **43,** 3175.

TWENTYMAN, P.R., KALLMAN, R.F. & BROWN, J.M. (1979). The effect of time between X-irradiation and chemotherapy on the growth of three solid mouse tumours. I. Adriamycin. *Int. J. Radiat. Oncol. Biol. Phys.,* **5,** 1255.

TWENTYMAN, P.R., BROWN, J.M., GRAY, J.W., FRANKO, A.J., SCOLES, M.A. & KALLMAN, R.F. (1980). A new mouse tumor model system (RIF-1) for comparison of end point studies. *J. Natl Cancer Inst.,* **64,** 595.

TWENTYMAN, P.R. & WORKMAN, P. (1983). An investigation of the possibility of chemosensitization by clinically achievable concentrations of misonidazole. *Br. J. Cancer,* **47,** 187.

WORKMAN, P., LITTLE, C.J., MARTEN, T.R. & 4 others. (1978). Estimation of the hypoxic cell sensitizer misonidazole and its 0-demethylated metabolite in biological materials by reversed-phase liquid chromatography. *J. Chromatogr.,* **145,** 507.

WORKMAN, P., TWENTYMAN, P.R., LEE, F.Y.F. & WALTON, M.I. (1983). Drug metabolism and chemosensitization. *Biochem. Pharmacol.,* **32,** 857.